# Quantification of Huntington’s Disease Related Markers in the R6/2 Mouse Model

**DOI:** 10.3389/fnmol.2020.617229

**Published:** 2021-01-11

**Authors:** Estibaliz Etxeberria-Rekalde, Saioa Alzola-Aldamizetxebarria, Stefanie Flunkert, Isabella Hable, Magdalena Daurer, Joerg Neddens, Birgit Hutter-Paier

**Affiliations:** ^1^QPS Austria GmbH, Grambach, Austria; ^2^Department of Health Studies, FH Joanneum University of Applied Sciences, Graz, Austria

**Keywords:** Huntington animal model, TSPO, histological evaluation, Ctip2, quantification

## Abstract

Huntington’s disease (HD) is caused by an expansion of CAG triplets in the huntingtin gene, leading to severe neuropathological changes that result in a devasting and lethal phenotype. Neurodegeneration in HD begins in the striatum and spreads to other brain regions such as cortex and hippocampus, causing motor and cognitive dysfunctions. To understand the signaling pathways involved in HD, animal models that mimic the human pathology are used. The R6/2 mouse as model of HD was already shown to present major neuropathological changes in the caudate putamen and other brain regions, but recently established biomarkers in HD patients were yet not analyzed in these mice. We therefore performed an in-depth analysis of R6/2 mice to establish new and highly translational readouts focusing on Ctip2 as biological marker for motor system-related neurons and translocator protein (TSPO) as a promising readout for early neuroinflammation. Our results validate already shown pathologies like mutant huntingtin aggregates, ubiquitination, and brain atrophy, but also provide evidence for decreased tyrosine hydroxylase and Ctip2 levels as indicators of a disturbed motor system, while vesicular acetyl choline transporter levels as marker for the cholinergic system barely change. Additionally, increased astrocytosis and activated microglia were observed by GFAP, Iba1 and TSPO labeling, illustrating, that TSPO is a more sensitive marker for early neuroinflammation compared to GFAP and Iba1. Our results thus demonstrate a high sensitivity and translational value of Ctip2 and TSPO as new marker for the preclinical evaluation of new compounds in the R6/2 mouse model of HD.

## Introduction

Huntington’s disease (HD) is an autosomal-dominant inherited neurodegenerative disorder that typically starts in the fourth decade of life (Conneally, 1984) and affects about 5–10 per 100,000 individuals in the Western European population (Morrison et al., 1995; Rawlins et al., 2016). The earliest symptoms include motor deficits, cognitive impairments, and neuropsychiatric dysfunctions in advanced disease stages (Kirkwood et al., 2001).

Huntington’s disease is caused by an expansion of CAG repeats in exon 1 of the human huntingtin gene (*HTT*) located on chromosome 4. It encodes the huntingtin protein, which is essential for a physiologically normal neuronal development (Ring et al., 2015). The expanded *HTT* sequence encodes for a mutated version of the huntingtin protein (mHTT), which causes conformational changes, leading to both cytosolic and nuclear aggregates and, thus, intracellular alterations, toxicity and neuronal loss (Landles et al., 2010; Illarioshkin et al., 2018).

For a better understanding of the disease, the first transgenic mouse models of HD named the R6/1 and R6/2 lines were developed in 1996. The original R6/1 model expresses the human *HTT* gene with approximately 115 CAG repeats while the original R6/2 model expresses about 150 CAG repeats (Mangiarini et al., 1996). R6/2 mice have a more severe phenotype and are characterized by progressive brain atrophy in the neostriatum and cerebral cortex, weight loss, motor dysfunction and neuronal accumulations of mHTT aggregates (Mangiarini et al., 1996; Carter et al., 1999; Stack et al., 2005). These mouse models are thus closely mimicking the human disease phenotype and are therefore thought to be the most suitable model to mimic HD and evaluate new drugs against this devastating disease (Menalled et al., 2009). Several years ago, the original R6/2 mouse line was shown to have a reduced number of about 120 CAG repeats. Animals presented with a slightly decreased disease severity and a delayed onset of the HD phenotype. Since then, both R6/2 sublines are available for HD research (Cowin et al., 2011).

The most prominent neuropathological finding of the disease is neuronal loss in selected brain regions. Neurodegeneration in HD begins in the striatum where 95% of neurons are GABAergic medium spiny neurons (MSNs) that are strongly reduced in late disease stages (Vonsattel et al., 1985; Arlotta et al., 2008). COUP-TF interacting protein 2 (Ctip2), also known as Bcl11b, is a transcription factor expressed in striatal MSNs (Desplats et al., 2008), in some areas of the hippocampus (Simon et al., 2012, 2016) and in cortical motoneurons (Leid et al., 2004; Arlotta et al., 2005; Tantirigama et al., 2016), and it was shown to bind mHTT protein in R6/1 and YAC72 transgenic HD mice (Desplats et al., 2008). The transcription factor is strongly reduced in HD cell lines, mouse models and patients (Desplats et al., 2008; Ahmed et al., 2015). Ctip2 might thus be a promising marker for HD.

Early neuronal injury leads to gliosis in many HD mouse models (Lin et al., 2001) and in brains of HD patients as analyzed *post mortem* (Sapp et al., 2001). Many imaging biomarkers to evaluate disease progression have been developed but so far only one biomarker was able to track a therapeutic response in animal models or HD patients. This C-(R)-PK11195 marker has been previously used for the assessment of disease state in HD patients by PET imaging labeling the translocator protein 18 kDa (TSPO) (Tai et al., 2007; Politis et al., 2008; Politis et al., 2011). TSPO is a mitochondrial membrane receptor that was associated with reactive microglia. However, its expression was also reported in astrocytes (Lavisse et al., 2012; Notter et al., 2018). In general, TSPO expression was observed to be upregulated in injured brain tissue and neuroinflammation (Simmons et al., 2018). Evaluating TSPO in HD would have a direct impact to understand its potential role as pharmacological target and its use in medical imaging.

Continued research toward developing biological markers to understand HD mechanisms that can track disease progression and therapeutic efficacy would be of great benefit for HD research. In this study, we therefore performed an in-depth qualitative and quantitative histological characterization of the brain of early and late symptomatic R6/2 mice with about 120 CAG repeats. Specifically, the cerebral cortex, hippocampus and caudate putamen were evaluated as these brain regions are of great interest as therapeutic target to improve motor and cognitive deficits in these mice. The occurrence of brain atrophy was measured, and immunofluorescent labeling was used to investigate ubiquitination and transgenic human mHTT levels in the nucleus and cytosol, to validate already well-characterized pathological features of R6/2 mice. We evaluated tyrosine hydroxylase (TH) and vesicular acetylcholine transporter (VAChT) levels as markers of the dopaminergic and cholinergic system, respectively, and Ctip2 as potentially new readout for HD. Finally, occurrence of astrogliosis and microgliosis were analyzed in distinct brain regions by Iba1 and GFAP labeling, respectively. Furthermore, Iba1 and GFAP labeling were compared to levels of the neuroinflammation marker TSPO. Hypothesizing that Ctip2 and TSPO are valuable and highly translational readouts for HD research, we investigated these readouts at different ages because this information on progression of the phenotype is crucial for planning and proper execution of preclinical studies testing the efficacy of new pharmacological treatments.

## Materials and Methods

### Animals

R6/2 mice and non-transgenic (ntg) littermates were purchased from Jackson Laboratories, United States (62Gpb/3J; #006494) and housed under identical conditions in individually ventilated cages on standard rodent bedding (Rettenmaier^®^, Germany) in the AAALAC-accredited animal facility of QPS Austria GmbH. Cotton nestlets (Plexx^®^) were provided as nesting material. The room temperature was kept at approximately 21°C and the relative humidity between 40 and 70%. Mice were housed in same sex groups of up to four animals under constant light-cycle (12 h light/dark). If animals had to be separated due to fighting, the single housed animal received wood wool as additional nesting material. Dried pelleted standard rodent chow (Altromin^®^, Germany) and normal tap water were provided *ad libitum*. Each individual animal was checked regularly for any clinical signs. Eight and 15 weeks old male and female animals of equal number were used. Actual animal numbers are given in the figure legends.

Analysis of number of CAG repeats showed that all R6/2 mice used in this study presented 121–127 CAG repeats.

Animal studies conformed to the Austrian guidelines for the care and use of laboratory animals (Tierversuchsgesetz 2012-TVG 2012, BGBl. I Nr. 114/2012). Animal housing and euthanasia were approved by the Styrian government (Amt der Steiermärkischen Landesregierung, Abteilung 13 – Umwelt und Raumordnung Austria; ABT13-78Jo115/2013-2016; ABT13-78Jo-118/2013-13).

### Tissue Sampling and Preparation

All mice were anesthetized by intraperitoneal injection of 600 mg/kg pentobarbital. Mice were then transcardially perfused with physiological (0.9%) saline. The right hemisphere was collected and immediately fixed in freshly prepared 4% paraformaldehyde in 0.1 M phosphate buffer (pH 7.4) for 2 hours (h) at room temperature (RT), subsequently cryo-protected in 15% sucrose/phosphate buffered saline (PBS; DPBS, PO4-360000, Pan Biotech, Aidenbach, Germany) solution over night at 4°C and then embedded in OCT medium and snap-frozen in liquid dry ice-cooled isopentane. 10 μm thick sagittal brain sections were systematically cut using a cryostat (CM 3050 S, Leica). For each immunofluorescent labeling, a total of 5 sections per animal from 5 different mediolateral levels throughout the whole hemisphere were used. Throughout the procedure sections were kept and washed in PBS, pH 7.4, at RT unless noted otherwise.

### Immunofluorescent Labeling of Huntingtin, Ubiquitin, and NeuN

Cryosections were air-dried for 45 min, washed in PBS, and then treated with 1x citrate buffer (AP-9003, Thermo Scientific, Waltham, United States) for 15 min at 95°C. After cooling to RT murine sections were blocked in M.O.M. Blocking Reagent (BMK-2202, Vector Laboratories, Inc., Burlingame, United States) in 0.1% Triton X-100/PBS for 1 h at RT. Afterward, tissue sections were incubated with primary antibodies (mouse anti-Huntingtin antibody, 1:250, Millipore MAB5374; guinea pig anti-NeuN antibody, 1:2000, Synaptic Systems 266004; rabbit anti-Ubiquitin antibody, 1:250, Abcam ab134953) over night at 4°C. The primary antibody binding was visualized by labeling with the corresponding fluorophore-conjugated secondary antibodies [anti-mouse IgG (H + L), DyLight 650, 1:500, Thermo Fisher Scientific SA5-10169; anti-guinea pig IgG (H + L), Cy3, 1:500, Jackson ImmunoResearch, 706-165-148; anti-rabbit IgG (H + L), DyLight 755, 1:500, Thermo Fisher Scientific, SA5-10043] for 1 h at RT. Cell nuclei were then labeled using a DAPI counterstaining (A1001, PanReac AppliChem GmbH, Darmstadt, Germany) and sections were subsequently washed in PBS and distilled water and covered with Mowiol (Sigma-Aldrich, St. Louis, MO, United States) and coverslips.

### Immunofluorescent Labeling of GFAP, Iba1, and TSPO

Cryosections were air-dried for 45 min, washed in PBS and then blocked in 10% donkey serum/0.1% Triton X-100/PBS for 1 h at RT. Afterward, tissue sections were incubated with primary antibodies (goat anti-GFAP antibody, 1:500, Abcam ab53554; guinea pig anti-Iba1 antibody, 1:2000, Synaptic Systems 234004; rabbit anti-TSPO/PBR antibody, 1:400, Abcam ab109497) over night at 4°C. The primary antibody binding was visualized by labeling with the corresponding fluorophore-conjugated secondary antibodies [anti-goat IgG (H + L) Alexa Fluor 750, 1:500, Abcam ab175745; anti-guinea pig IgG (H + L), Cy3, 1:500, Jackson ImmunoResearch, 706-165-148; anti-rabbit IgG (H + L), DyLight 650, 1:500, Abcam, ab96922] for 1 h at RT. Cell nuclei were then labeled using DAPI counterstaining (A1001, PanReac AppliChem GmbH, Darmstadt, Germany) and sections were washed in PBS and distilled water and covered with Mowiol (Sigma Aldrich, St. Louis, MO, United States) and coverslips.

### Immunofluorescent Labeling of TH, VAChT, and Ctip2

Cryosections were air-dried for 45 min, washed in PBS and then treated with 1× citrate buffer (AP-9003, Thermo Scientific, Waltham, MA, United States) for 15 min at 95°C. After cooling to RT autofluorescence was reduced with sodium borohydride (213462, Sigma-Aldrich) for 4 min. Then, the sections were blocked in 10% donkey serum/0.1% Triton X-100/PBS for 1 h at RT. Afterward, tissue sections were incubated with primary antibodies (rat anti-Ctip2 antibody, 1:2000, Abcam ab18465; sheep anti-TH antibody, 1:500, Novus Biologicals NB300-110; guinea pig anti-VAChT antibody, 1:500, Synaptic Systems, 139105) over night at 4°C. The primary antibody binding was visualized by labeling with the corresponding fluorophore-conjugated secondary antibodies [anti-rat IgG (H + L), Alexa Fluor 488, 1:500, Jackson ImmunoResearch, 712-545-153; anti-goat IgG (H + L) Alexa Fluor 750, 1:500, Abcam ab175745; anti-guinea pig IgG (H + L), Cy3, 1:500, Jackson ImmunoResearch, 706-165-148] for 1 h at RT. Cell nuclei were then labeled using DAPI counterstaining (A1001, PanReac AppliChem GmbH, Darmstadt, Germany) and sections were washed in PBS and distilled water and covered with Mowiol (Sigma Aldrich, St. Louis, MO, United States) and coverslips.

In all three incubations, tissue sections were washed with PBS for 5 min at least twice between experimental steps. All steps during and after the use of fluorophore-conjugated antibodies were performed in the dark.

### Imaging and Quantitative Image Analysis

Mosaic images of the immunofluorescently labeled sections were recorded on a Zeiss automatic microscope AxioScan Z1 with high aperture lenses, equipped with a Zeiss Axiocam 506 mono and a Hitachi 3CCD HV-F202SCL camera and Zeiss ZEN 2.3 software. Quantitative image analysis was performed with Image Pro 10 (Media Cybernetics). The cerebral cortex, hippocampus, caudate putamen, and substantia nigra were identified and their size determined by drawing the region of interest (ROI) on the images. Background correction was used if necessary, and the immunofluorescence signal was then macro-based and thus rater-independently quantified by adequate thresholding and morphological filtering (size and shape) to determine the percentage of ROI area covered by immunoreactive (IR) objects, so that the resulting values are normalized for ROI size. After defining the parameters for detection of the targeted objects, image analysis was performed automatically using the same parameters on all images.

The numerical density (n/mm^2^) of nuclear and cytosolic mHTT inclusions was evaluated in the cortex, hippocampus and caudate putamen. Nuclear mHTT density was evaluated by counting mHTT inclusions overlapping with nuclear DAPI staining whereas cytosolic mHTT density was evaluated by counting mHTT inclusions within NeuN signal that did not overlap with DAPI staining. Data were then normalized by setting the group means of ntg animals to zero.

### Statistics

Data analysis was performed in GraphPad Prism^TM^ 8.1 (GraphPad Software Inc., United States). Graphs show group means and standard error of the mean (SEM). The significance level was set at *p* < 0.05. Group means were compared using Two-way analysis of variance (ANOVA) with a subsequent *post hoc* test. The utilized statistical tests and exact sample numbers are mentioned in the figure legends.

### Raw Data

Raw data of all quantifications are provided in Supplementary Table 1.

## Results

### Huntingtin Aggregates in R6/2 Mice

**FIGURE 1:**
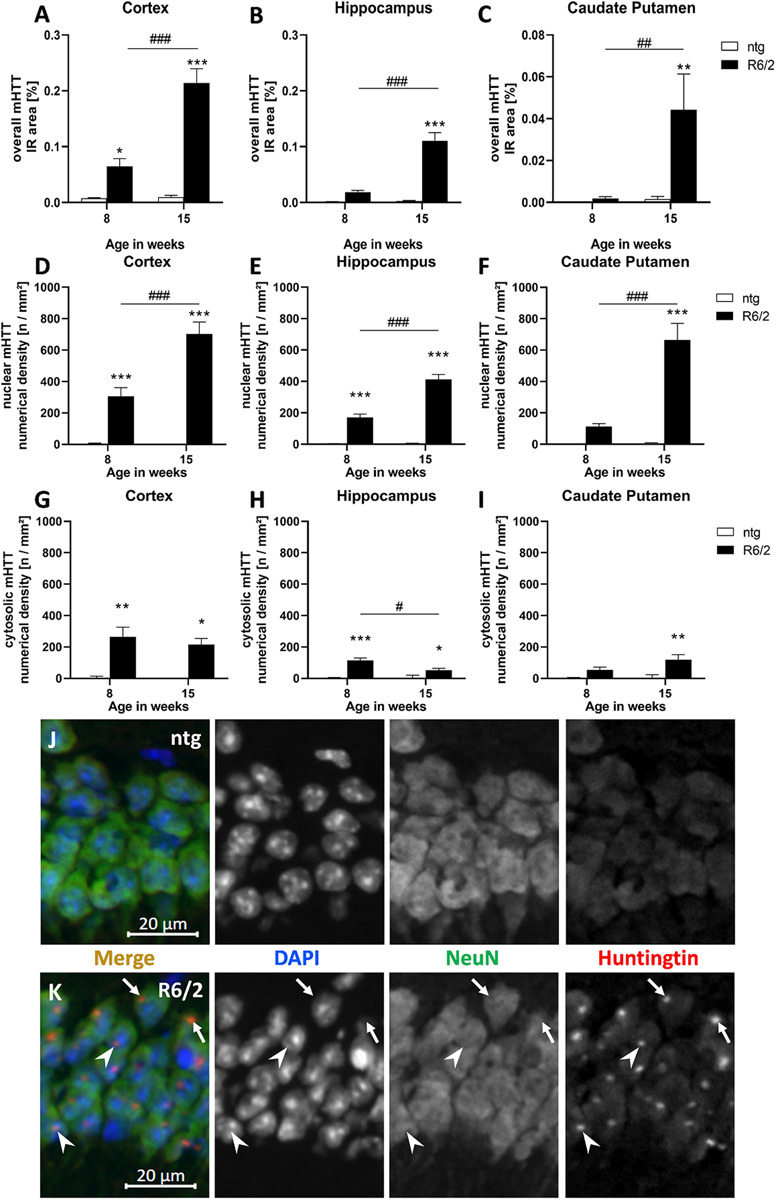
Quantification of overall mHTT as well as nuclear and cytosolic mHTT aggregates in R6/2 mice. The cortex **(A,D,G)**, hippocampus **(B,E,H)**, and caudate putamen **(C,F,I)** of 8 and 15 weeks old R6/2 mice was analyzed for overall mHTT immunoreactive (IR) area in percent **(A–C)** as well as the number of nuclear **(D–F)** and cytosolic **(G–I)** mHTT aggregates per mm^2^ compared to ntg littermates; *n* = 8 per group. Mean + SEM; Two-way ANOVA followed by Bonferroni’s *post hoc* test; *p < 0.05, **p < 0.01, ***p < 0.001. *Differences between genotypes; ^#^differences between age groups. Immunofluorescence in the hippocampal CA1 pyramidal cell layer **(J,K)** shows occurrence of mHTT aggregates both in the cytosol (arrows) and in nuclei (arrowheads) of R6/2 mice; note absence of aggregates in ntg tissue.

To evaluate overall mHTT levels, the immunoreactive (IR) area of human mHTT in the cortex, hippocampus, and caudate putamen of R6/2 and ntg mice was analyzed at the age of 8 and 15 weeks. This analysis revealed that immunoreactivity of human-specific mHTT was several fold higher (statistically significant in all three regions) in 15 weeks old R6/2 mice compared to ntg controls that showed only minor background signal (Figures 1A–C). In contrast, immunofluorescent signal was much lower at age 8 weeks, so the amount of mHTT significantly increased with age in all three brain regions (230% in the cortex, 497% in the hippocampus, 2,250% in the caudate putamen; Figures 1A–C).

In a next step, we evaluated the numerical density (n/mm^2^) of nuclear versus cytosolic mHTT aggregates. The occurrence of nuclear mHTT showed a very similar increase than overall mHTT, as the density of nuclear aggregates significantly increased with age in all analyzed brain regions (Figures 1D–F). In contrast, the density of cytosolic mHTT aggregates (Figures 1G–I) was much lower compared to nuclear mHTT aggregates and did not increase with age but rather decreased, especially in the hippocampus (Figure 1H).

Representative images of double labeling of mHTT and NeuN in combination with DAPI staining show nuclear (arrowheads) and cytosolic (arrows) mHTT aggregates in R6/2 compared to ntg mice (Figures 1J,K).

### Ubiquitination in R6/2 Mice

**FIGURE 2:**
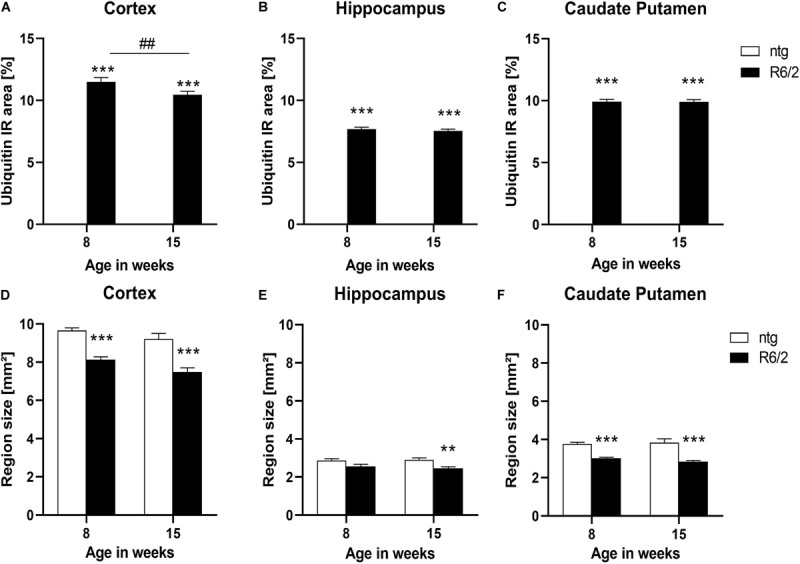
Quantification of ubiquitination and brain size alterations in R6/2 mice. The cortex **(A,D)**, hippocampus **(B,E)** and caudate putamen **(C,F)** of R6/2 mice was analyzed for ubiquitin **(A–C)** immunoreactive area (IR) in percent as well as region size **(D–F)** at the age of 8 and 15 weeks compared to ntg littermates; *n* = 8 per group. Mean + SEM; Two-way ANOVA followed by Bonferroni’s *post hoc* test; *p < 0.05, **p < 0.01, ***p < 0.001. *Differences between genotypes; #differences between age groups.

As the appearance of huntingtin aggregates often precedes ubiquitination, we analyzed ubiquitin expression in the cortex, hippocampus, and caudate putamen of R6/2 and ntg mice, focusing on these aggregates. While there were no aggregates in ntg mice resulting in low ubiquitin signal at either 8 and 15 weeks, R6/2 mice showed considerable signal at both ages that were several hundred fold higher compared to ntg mice (Figures 2A–C). Ubiquitin signal was rather stable during aging except for a small but significant decrease in the cortex of R6/2 mice (−9%; Figure 2A).

### Brain Atrophy in R6/2 Mice

Since R6/2 mice are known to display brain atrophy, the size of different brain regions of 8 and 15 weeks old R6/2 and ntg mice was measured. The results show that the size of the cortex and caudate putamen of R6/2 mice was significantly decreased at the age of 8 and 15 weeks compared to ntg animal (cortex: −15% at the age of 8 weeks and −18% at the age of 15 weeks; caudate putamen: −19% at the age of 8 weeks and −25% at the age of 15 weeks; Figures 2D,F), while in the hippocampus a significant decrease was observed only at the age of 15 weeks (−10% at the age of 8 weeks and −15% at the age of 15 weeks; Figure 2E). Analysis of R6/2 and ntg mice with age showed no significant changes in region size.

**FIGURE 3:**
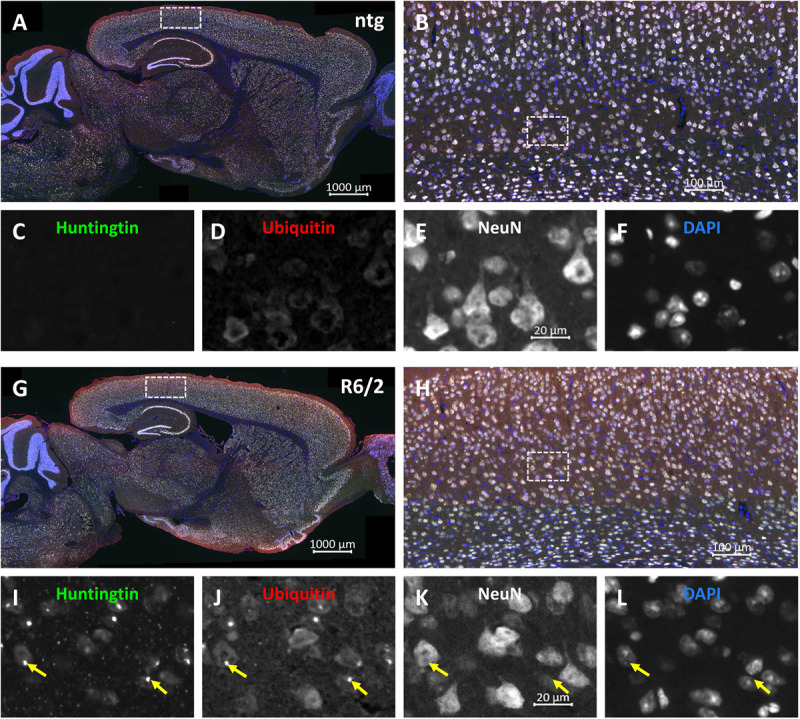
Representative images of huntingtin, ubiquitin and NeuN labeling of a brain of a R6/2 mouse. Immunofluorescent labeling of huntingtin (**G–I**; green), ubiquitin (**G,H,J**; red) and NeuN (**G,H,K**; white) in a 15 weeks old R6/2 mouse compared to an age-matched ntg littermate **(A–F)**. Magnified areas in **(B,H)** show cortex as indicated in the whole brain image **(A,G)**. Nuclei are labeled with DAPI (**F,L**; blue). Yellow arrows point toward accumulations of huntingtin and ubiquitin in both neuronal and non-neuronal cells **(I–L)**. Scale bars: **(A,G)** 1,000 μm. **(B,H)** 100 μm. **(C–F,I–L)** 20 μm.

Representative images of triple labeling of mHTT, ubiquitin and NeuN show very strong expression of ubiquitin and mHTT in the cortex of a 15 weeks old R6/2 mouse compared to a ntg mouse (Figure 3). Expression of ubiquitin was most intense on mHTT-positive aggregates within neuronal cells. Although mHTT and ubiquitin were quantified in cortex, hippocampus and caudate putamen only, expression levels of both markers were very high throughout the brain.

### Motor Function-Related Proteins

**FIGURE 4:**
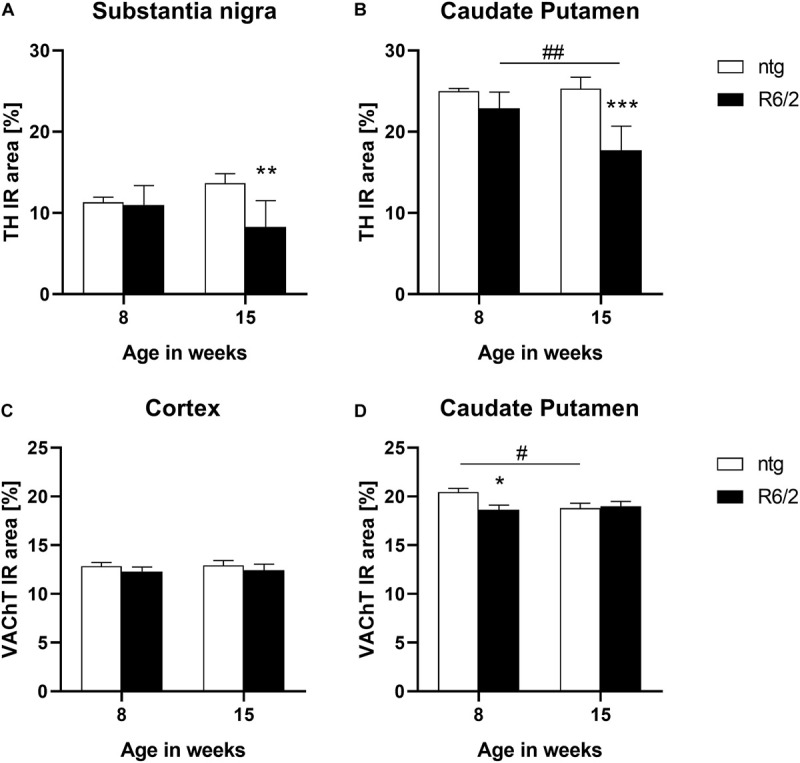
Quantification of TH and VAChT in early and late symptomatic R6/2 mice. The substantia nigra and caudate putamen of R6/2 mice was analyzed for TH **(A,B)** and the cortex and caudate putamen of R6/2 mice was analyzed for VAChT **(C,D)** immunoreactive area (IR) in percent at the age of 8 and 15 weeks compared to ntg littermates; *n* = 8 per group. Mean + SEM; Two-way ANOVA with Bonferroni’s *post hoc* test; *p < 0.05, **p < 0.01, ***p < 0.001. *Differences between genotypes; ^#^differences between age groups.

In a next step TH levels were analyzed in target areas of the nigrostriatal system, the substantia nigra and caudate putamen. The results show that at the age of 15 weeks R6/2 mice presented significantly lower levels of TH in both brain regions (substantia nigra: −3% at the age of 8 weeks and −39% at the age of 15 weeks; caudate putamen: −8% at the age of 8 weeks and −30% at the age of 15 weeks; Figures 4A,B). Additionally, in the caudate putamen of R6/2 mice the levels of TH significantly decreased with age (caudate putamen: −22%; Figure 4B).

The expression of the vesicular acetylcholine transporter VAChT was analyzed by quantification of the IR of two different brain regions of R6/2 mice. In the cortex, no differences between R6/2 and ntg mice could be observed (−4% at the age of 8 weeks and −4% at the age of 15 weeks; Figure 4C), while a significant reduction of VAChT in the caudate putamen of R6/2 mice at the age of 8 weeks compared to ntg mice could be measured (−8% at the age of 8 weeks and −0.9% at the age of 15 weeks; Figure 4D). Ntg littermates showed a significant reduction of VAChT levels at the age of 15 weeks compared to 8 weeks (−8%; Figure 4D).

**FIGURE 5:**
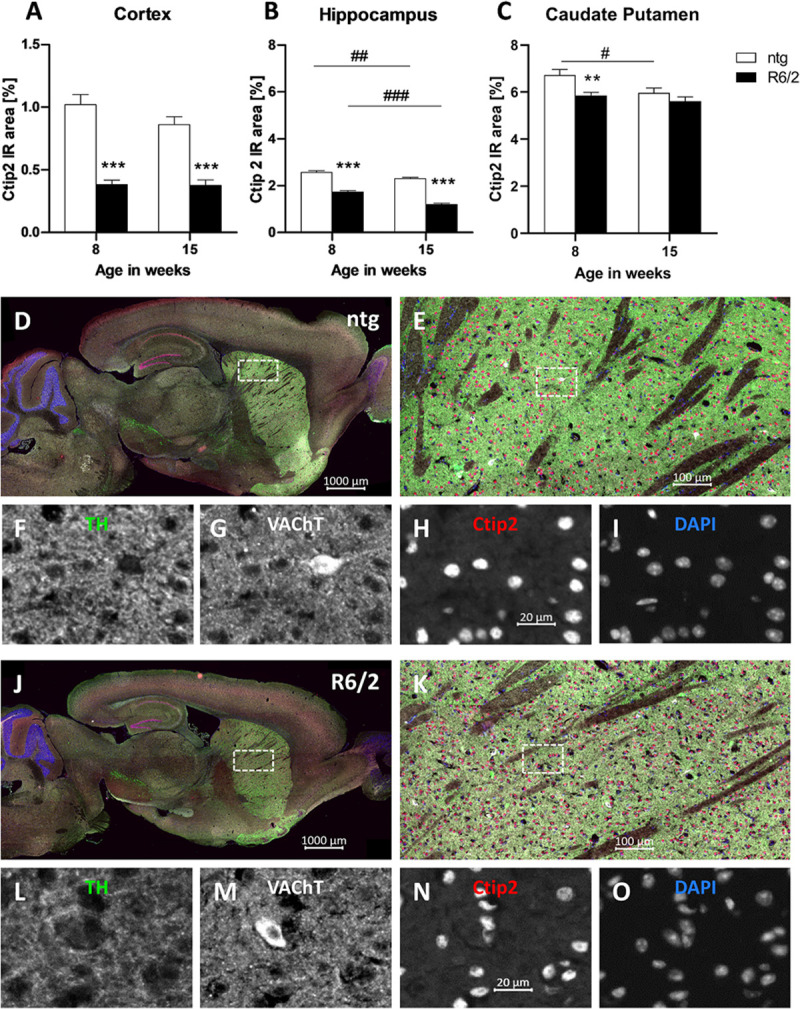
Quantification of Ctip2 in early and late symptomatic R6/2 mice and representative images of TH, VAChT and Ctip2 labeling. The cortex **(A)**, hippocampus **(B)**, and caudate putamen **(C)** of R6/2 mice was analyzed for Ctip2 immunoreactive area (IR) in percent at the age of 8 and 15 weeks compared to ntg littermates; *n* = 8 per group. Mean + SEM; Two-way ANOVA with Bonferroni’s *post hoc* test; *p < 0.05, **p < 0.01, ***p < 0.001. *Differences between genotypes; ^#^differences between age groups. Representative images of TH, VAChT and Ctip2 in the brain of a 15 months old R6/2 mouse are shown in **(D–O)**. Immunofluorescent labeling of TH (**J–L**; green), VAChT (**J,K,M**; white), and Ctip2 (**J,K,N**; red) in a 15 weeks old R6/2 mouse compared to an age-matched ntg littermate **(D–I)**. Magnified areas in **(E,K)** show caudate putamen as indicated in the whole brain image **(D,J)**. Nuclei are labeled with DAPI (**I,O**; blue). Scale bar: **(D,J)** 1,000 μm. **(E,K)** 100 μm. **(F–I,L–O)** 20 μm.

Furthermore, the IR of the transcription factor Ctip2 was quantitatively analyzed. Results show a significant decrease of the IR in the cortex (−62% at the age of 8 weeks and −56% at the age of 15 weeks; Figure 5A) and hippocampus (−32% at the age of 8 weeks and −47% at the age of 15 weeks; Figure 5B) of R6/2 mice at the age of 8 and 15 weeks compared to ntg mice and in the caudate putamen at the age of 8 weeks (−12% at the age of 8 weeks and −5% at the age of 15 weeks; Figure 5C). Furthermore, the IR area of Ctip2-positive objects in the hippocampus of R6/2 mice and in the hippocampus and caudate putamen of ntg mice decreased from week 8 to week 15 (cortex: −1.9%; hippocampus: −30%; caudate putamen: −4%; Figures 5B,C). However, only minor changes occurred in the caudate putamen, namely, a significant difference in 8 weeks old mice (−11% in R6/2 mice) that was not detectable any more at 15 weeks.

Representative images of the triple labeling of TH, VAChT, and Ctip2 validated the strong decrease in the expression of Ctip2 and a weaker decrease of VAChT and TH in 15 weeks old R6/2 mice compared to ntg littermates (Figures 5D–O). High TH expression was further found in the ventral tegmental area and olfactory tubercle, while high VAChT expression was additionally found in the brainstem. Strong Ctip2 expression was further found in the CA1 and CA2 region as well as the dentate gyrus and the subiculum. Weaker Ctip2 expression was observed in cortical pyramidal cells of layer 5 and 6 as well as the olfactory bulb.

### Neuroinflammation in R6/2 Mice

**FIGURE 6:**
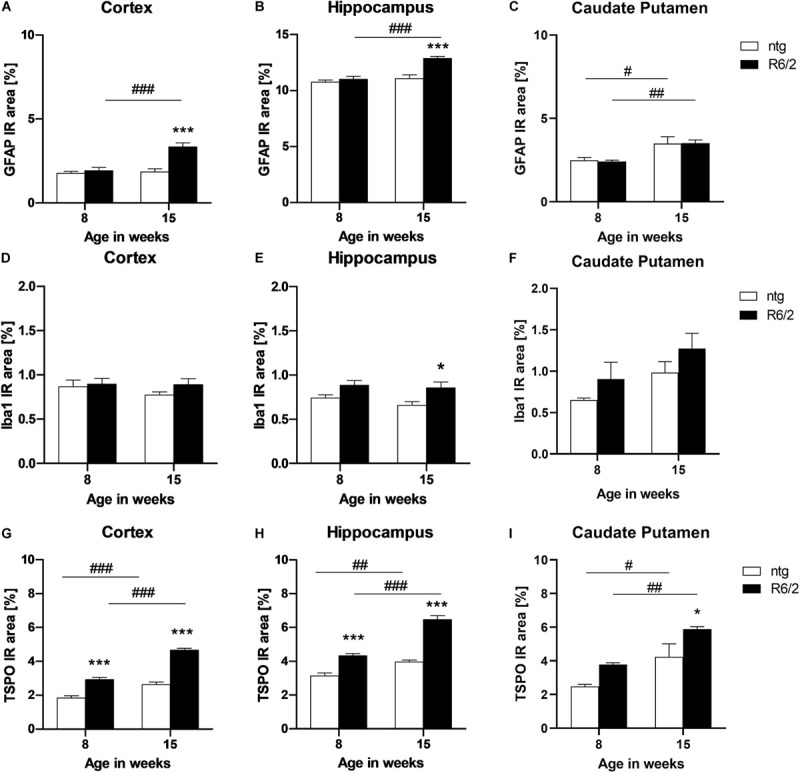
Quantification of astrocytosis by GFAP and activated microglia by Iba1 and TSPO labeling in R6/2 mice. The cortex **(A,D,G)**, hippocampus **(B,E,H)**, and caudate putamen **(C,F,I)** of R6/2 mice was analyzed for GFAP **(A–C)**, Iba1 **(D–F)**, and TSPO **(G–I)** immunoreactive area (IR) in percent at the age of 8 and 15 weeks compared to ntg littermates; *n* = 8 per group. Mean + SEM; Two-way ANOVA with Bonferroni’s *post hoc* test; *p < 0.05, **p < 0.01, ***p < 0.001. *Differences between genotypes; ^#^differences between age groups.

Quantification of the immunofluorescent signal of GFAP and Iba1 as a marker for reactive astrocytes and activated microglia, respectively, was performed in the cortex, hippocampus, and caudate putamen of early and late symptomatic R6/2 mice and corresponding ntg littermates. Significantly increased levels of GFAP in R6/2 mice compared to ntg animals were found at 15 weeks of age in the cortex and hippocampus (cortex: +81%; hippocampus: +16%; Figures 6A,B). In all three brain regions an age-dependent increase of GFAP signal could be observed in R6/2 mice (cortex: +73%; hippocampus: +17%; caudate putamen: +45%; Figures 6A–C). A region-specific increase of GFAP signal in the older ntg mice was detected in the caudate putamen (+40%; Figure 6C).

Analysis of Iba1 IR in the cortex and caudate putamen resulted in no significant difference between genotypes or age groups (Figures 6D,F), whereas in the hippocampus, Iba1 levels were significantly increased in R6/2 mice at 15 weeks of age compared to age-matched ntg littermates (+29%; Figure 6E).

Quantification of TSPO detected significantly higher IR area in all investigated brain regions of R6/2 mice (cortex: +57% at 8 weeks and +77% at 15 weeks; hippocampus: +38% at 8 weeks and +63% at 5 weeks; caudate putamen: +38% at 15 weeks; Figures 6G–I). In addition, TSPO levels significantly increased during aging in both R6/2 (cortex: +59%, hippocampus: +49%, caudate putamen: +55%; Figures 6G–I) and ntg mice (cortex: +42%, hippocampus: +27%, caudate putamen: +71%; Figures 6G–I).

**FIGURE 7:**
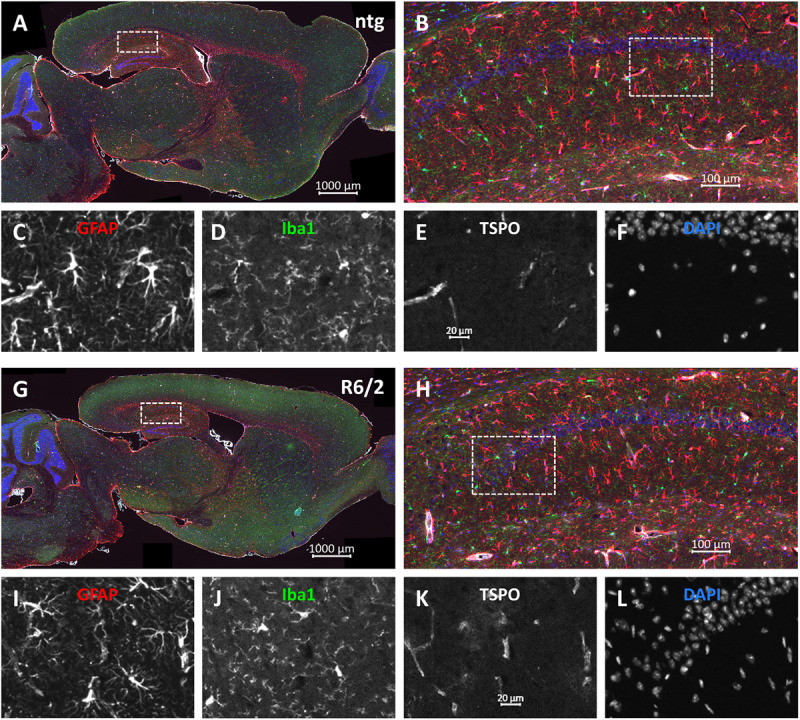
Representative images of GFAP, Iba1 and TSPO in the brain of a 15 months old R6/2 mouse. Immunofluorescent labeling of GFAP (**G–I**; red), Iba1 (**G,H,J**; green), and TSPO (**G,H,K**; white) in a 15 weeks old R6/2 mouse compared to an age-matched ntg littermate **(A–F)**. Magnified areas in **(B,H)** show hippocampus as indicated in the whole brain image. Nuclei are labeled with DAPI (**F,L**; blue). Scale bar: **(A,G)** 2.000 μm. **(B,H)** 200 μm. **(C–F,I–L)** 50 μm.

Representative images of the triple labeling of GFAP, Iba1 and TSPO show the patterns and differences in the hippocampus of R6/2 mice compared to ntg littermates (Figure 7). In addition to the quantitatively analyzed brain regions, strong GFAP expression was observed in the corpus callosum, olfactory bulb, and cerebellum while most other brain regions showed moderate GFAP expression. Additionally, strong TSPO expression was observed in the hypothalamus.

## Discussion

In this study we performed a histopathological characterization of specific brain regions of early and late symptomatic R6/2 mice with about 120 CAG repeats to gain a better understanding of the disease pathology and progression. First, commonly used markers for the pathology of R6/2 mice were used to validate the neuropathological phenotype of this mouse model. Second, to evaluate motor-related pathologies, we used an antibody against Ctip2, while VAChT was used as cholinergic neuron marker and TH as marker for monoaminergic neurons and especially the dopaminergic system. Third, we evaluated the neuroinflammatory component of the disease by using GFAP as indicator of astrocytosis and Iba1 as a microglial marker. Furthermore, expression of TSPO in R6/2 mice was evaluated as a marker for neuroinflammation with translational value.

### Huntingtin Aggregates and Ubiquitination

A neuropathological hallmark of HD found in human *post mortem* brains and in transgenic HD animal models is the accumulation of mHTT forming intracellular aggregates (Juenemann et al., 2011). These aggregates accumulate in MSNs of the striatum and in the cerebral cortex and appear before any sign of degeneration can be detected (Gutekunst et al., 1999). Our results validate that there is a significant increase of mHTT with age in R6/2 mice with about 120 CAG repeats, as already shown by several other groups for the original R6/2 mouse line with higher CAG repeat numbers (Orth et al., 2003; Gong et al., 2012). Gong et al. (2012) for example show that ubiquitinated mHTT inclusions can be found in the cortex, hippocampus and caudate putamen of R6/2 mice as early as 4 weeks of age in R6/2 mice with about 188 CAG repeats suggesting that this is a very early event in this model. mHTT aggregates are of high importance for the development of disease pathology, as it is supposed that a reduction of mHTT aggregates can improve striatal function (Valdeolivas et al., 2015). An analysis by Cummings et al. (2012) shows, that the severity of the HD phenotype and pathology strongly depends on the number of CAG repeats with the strongest effects of approximately 160 repeats. Utilizing R6/2 mice with a lower CAG repeat number of about 120 should thus result in a weaker or delayed phenotype onset as observed in the here presented study.

The detailed analysis of mHTT localization in R6/2 mice revealed that most aggregated mHTT can be found in the nucleus of cortical and caudate putamen tissue. These results confirm previous data on inclusions preferentially being localized in the nucleus that were obtained from R6/2 mice with a comparable number of CAG repeats as used in the present study (Davies et al., 1997). Our results further show that nuclear mHTT aggregates preferably occur in the cortex and caudate putamen, consistent with human disease where HTT aggregates are found in all cortical layers and the striatum, while other brain regions are barely affected (DiFiglia et al., 1997). The pathological effect of mHTT aggregates seems to be dependent on the nuclear localization of aggregates, because preventing the translocation of HTT fragments into the nucleus improved motor function and reduced atrophy (Cho et al., 2009).

We further show here that mHTT aggregates in R6/2 mice are highly ubiquitinated already at early symptomatic stage while ubiquitin levels are much lower in wild type animals and aggregate-like punctate signal is missing entirely. It was already shown that impairments in the ubiquitin proteasome system (UPS) are an additional feature of HD, since R6/2 mice with about 195 CAG repeats and HD patients show increased levels of polyubiquitin conjugates co-localized to mHTT aggregates (DiFiglia et al., 1997; Bennett et al., 2007). The UPS is a two-step process: First, it targets proteins to the polyubiquitin chain and second, it destroys the ubiquitylated proteins by a proteasome [for review see Huibregtse (2005)]. Inhibiting this process in HD causes accumulation of toxic mHTT protein fragments (Li et al., 2010). Details about the effects of ubiquitination on aggregation of mHTT and subsequent cellular responses were just recently reported (Hakim-Eshed et al., 2020).

### Brain Atrophy

In the present study we found evidence for a decreased size of the cortex, hippocampus and caudate putamen of 8 weeks old R6/2 mice and thus very early in the course of disease progression. Our results further strengthen results by Bissonnette et al. (2013), who showed that 10 weeks old R6/2 mice with the same number of CAG repeats as used here, have a reduced brain region size, particularly in the caudate putamen and neocortex compared to age-matched ntg littermates.

Zhang et al. (2010) revealed that brain atrophy in R6/2 mice is already detectable as early as 3 weeks of age and that it is more widely distributed in these mice compared to the human pathology, probably due to the more aggressive phenotype of this short fragment HD mouse model. This result is quite surprising considering that the group used a R6/2 line with only 103-112 CAG repeats (Zhang et al., 2010). How the mutation influences brain atrophy is still unclear, although it has been demonstrated that the intensity and rate of progression of brain atrophy are more pronounced in patients with larger, expanded CAG repeat sequences (Ruocco et al., 2008).

By evaluating the temporal expression profile of mHTT and ubiquitin as well as validating brain atrophy in early symptomatic R6/2 mice, we were thus able to validate the main pathological features of R6/2 mice and the robustness of these pathologies.

### Motor-Related Markers

Motor deficits are a well-described phenotype of the original R6/2 mouse line (Carter et al., 1999; Stack et al., 2005) but also of the here utilized line with about 120 CAG repeats (Reiner et al., 2012; Bissonnette et al., 2013). To evaluate the underlying neuropathological changes of these behavioral deficits, dopaminergic and cholinergic system markers were used.

It has been suggested that a dysfunction of dopaminergic neurotransmission can be relevant for the development of motor symptoms in HD (Johnson et al., 2006). Transgenic HD mouse models, including the original R6/2 mouse line, thus show a reduction of dopamine (DA) in the caudate putamen in the late stages of the disease (Callahan and Abercrombie, 2011). Here, we show a reduced expression of TH, the rate-limiting enzyme in DA production, in the substantia nigra and caudate putamen of R6/2 mice. Our results therefore validate previous results about pathological changes in the dopaminergic system of R6/2 mice. Since Bedard et al. (2011) demonstrated a significant decrease of TH immunoreactivity in the *post mortem* HD striatum, the mouse model closely mimics the pathological changes in the dopaminergic system of the human disease.

Another prominent feature of the original R6/2 mouse line is a progressive striatal cholinergic dysfunction causing movement disturbances (Farrar et al., 2011). As acetylcholine plays a key role in cholinergic neurotransmission, we analyzed the expression of VAChT as marker of presynaptic cholinergic synapses and found levels to be decreased in the caudate putamen of R6/2 mice. de Castro et al. (2009) previously described that a reduction in the expression levels of this transporter in VAChT knockdown [KD^HOM^] mice leads to myasthenia and cognitive deficits. Reduced VAChT levels in the R6/2 mouse caudate putamen might hence be causative for the development of motor deficits.

Since HD pathology is characterized by MSN death in the striatum (Albin et al., 1992), we evaluated the expression of Ctip2, a protein expressed in striatal MSNs and required for the correct development of these cells (Arlotta et al., 2008). Additionally, Ctip2 is expressed in the hippocampus and cortical GABAergic interneurons (Arlotta et al., 2005) and important for physiological adult neurogenesis in the hippocampal dentate gyrus (Simon et al., 2012). Fjodorova et al. (2019) demonstrated that Ctip2 plays a neuroprotective role against oxidative stress and that it is essential for maintaining striatal MSN characteristics. Our results show a reduction of the intensity of Ctip2 labeling in the cortex, hippocampus, and in young animals also the caudate putamen; suggesting that hypofunction of Ctip2 might contribute to the development of brain atrophy in R6/2 mice. Due to the strong and early decrease of Ctip2 levels in the cortex and hippocampus of R6/2 mice, this protein might be a valuable biological marker for early HD diagnosis.

### Neuroinflammation in R6/2 Mice

Although cell-autonomous pathogenesis is a central feature of HD, non-cell-autonomous pathogenesis is increasingly recognized. Glial cells play a key role in this process, as the expression of mHTT in astrocytes produces a neurological phenotype in HD knock-in mice (Bradford et al., 2009). Moreover, a recent study confirms that activated microglia are present in early symptomatic HD (Tai et al., 2007).

In a first analysis we show a significant increase of astrocytosis in the cortex and hippocampus but not the caudate putamen of symptomatic R6/2 mice measured by GFAP labeling. These results are consistent with a previous analysis that showed increased GFAP levels in the cerebellum and cortex but not the caudate putamen of R6/2 mice by Western blotting (Luthi-Carter et al., 2002). In HD patients, recent findings suggest that the size of astrocytes increases with HD progression (Faideau et al., 2010). Astrocytosis as observed in R6/2 mice is thus a readout with high translational value.

Under physiological conditions, microglia remove dead cells from the brain (Wu et al., 2014). However, activated microglia can also cause neuronal death since they release a variety of immune cytokines and neurotoxic factors, causing neurodegeneration [for review see Brown and Vilalta (2015)].

In this study we evaluated Iba1 levels in the early and late symptomatic brain of R6/2 mice. Iba1 as a marker for activated microglia (Ohsawa et al., 2004) has been used to highlight the ramification of microglia cells (Hirayama et al., 2001). We found a general tendency of somewhat higher Iba1 immunoreactivity in R6/2 mice, which was significant in the hippocampus of 15 weeks old mice. There are several factors that may contribute to this difference, such as larger size of microglia, increased Iba1 expression, and a higher number of activated microglia (Kozlowski and Weimer, 2012; Avignone et al., 2015; Davis et al., 2017). In fact, our panel of quantitative readouts generated during image analysis revealed that all these factors contribute to the differences in the percentage of immunoreactive area presented in Figure 6. Our quantitative results are similar to published data, showing that activated microglia can be found as early as 11 weeks of age in the caudate putamen of R6/2 mice and also in the striatum of early stage HD patients, further increasing with age (Simmons et al., 2007, 2016). Data on microglia in R6/2 mice are a controversially discussed topic, as results on Iba1 levels in the caudate putamen at different stages of the disease are difficult to interpret (Simmons et al., 2007). A recent study by Yu-Taeger et al. (2019), found no significant changes in Iba1 expression levels in the caudate putamen of 11.5 weeks old R6/2 mice. However, one research group found a decrease in the number of Iba1 positive cells in the cortex of 14.5–16 weeks old R6/2 mice while others found an increase in Iba1 cell density in the striatum at 4 and 13 weeks of age and also an increase in the Iba1 positive area (Ma et al., 2003; Paldino et al., 2020a,b).

Additionally, we evaluated expression levels of TSPO, a mitochondrial membrane receptor that is expressed in activated microglia and reactive astrocytes (Betlazar et al., 2018). So far, TSPO is mostly used for the diagnosis of HD in humans. While its expression levels are low under physiological conditions, TSPO is upregulated during neuroinflammation (Veiga et al., 2007). Recent PET studies using a TSPO tracer determined that microglia are activated in the striatum and cortex but not the hippocampus of presymptomatic and symptomatic HD patients. However, similar to our results, the accumulation of TSPO in R6/2 mice with about 120 CAG repeats was also evident in the hippocampus (Simmons et al., 2018). These results suggest that microglial activation is progressively increasing in the striatum and cortex of HD patients and mouse models in early stages of the disease.

Iba1 and GFAP are markers for microglia and astrocytes, respectively, and TSPO seems to be a general marker of early neuroinflammation being expressed in some activated microglia and a subset of astrocytes. However, we observed quantitative differences in IR area levels between these markers. TSPO immunoreactivity significantly increased at the age of 8 weeks in all three investigated regions, cortex, hippocampus, and caudate putamen. In contrast, differences of Iba1 immunoreactivity occurred only in the hippocampus and GFAP immunoreactivity in the cortex and hippocampus. The different labeling patterns lead us to hypothesize that TSPO is a more sensitive marker of early neuroinflammation, as it is able to detect the activity level of mitochondria in neuroinflammatory cells (Lejri et al., 2019), although it does not distinguish between different neuroinflammatory cell types. In parallel, Iba1 is expressed at a later stage, when the morphological change to ameboid shape of microglia is already accomplished as it is involved in membrane ruffling and phagocytosis of activated microglia (Ohsawa et al., 2000). Due to the high sensitivity of TSPO for the recognition of neuroinflammatory processes, TSPO might be a highly valuable and translatable readout for clinical and preclinical HD research.

## Conclusion

The aim of this study was to establish new and highly translational biological markers to label motor system-related cells and neuroinflammation in R6/2 mice. Our analysis suggests that Ctip2 might be a valuable readout for the early detection of HD pathological changes. Furthermore, our study shows somewhat higher levels of GFAP and Iba1, while TSPO may be a more sensitive biological marker of neuroinflammation in R6/2 mice. These findings validate the highly translational phenotype of R6/2 mice compared to pathological alterations described for HD patients, and further highlight the merit of Ctip2 and TSPO as translational readouts for HD research. Ultimately, these results will not only help to gain a better understanding of HD pathogenesis but will also allow a more efficient HD compound testing.

## Data Availability Statement

The original contributions generated for this study are included in the article/Supplementary Material, further inquiries can be directed to the corresponding author.

## Ethics Statement

Animal studies conformed to the Austrian guidelines for the care and use of laboratory animals (Tierversuchsgesetz 2012-TVG 2012, BGBl. I Nr. 114/2012). Animal housing and euthanasia were approved by the Styrian government (Amt der Steiermärkischen Landesregierung, Abteilung 13 – Umwelt und Raumordnung Austria; ABT13-78Jo115/2013-2016; ABT13-78Jo-118/2013-13).

## Author Contributions

EE-R planned, performed, and analyzed histological labelings and edited the manuscript. SA-A and SF prepared figures and wrote the manuscript. IH performed and analyzed histological labelings. MD supervised parts of the histological evaluations and edited the manuscript. JN planned and analyzed histological labelings and edited the manuscript. BH-P conceived the whole study and edited the manuscript. All authors contributed to the article and approved the submitted version.

## Conflict of Interest

EE-R, SF, MD, JN, and BH-P are employees of QPS Austria GmbH. SA-A was employed as an intern by QPS Austria GmbH while the research was conducted. IH was a (unpaid) collaborator while carrying research for her BSc thesis at QPS Austria GmbH.

## Abbreviations

AAALAC: Association for Assessment and Accreditation of Laboratory Animal Care
ANOVA: analysis of variance
CNS: central nervous system
CSF: cerebrospinal fluid
Ctip2: COUP-TF interacting protein 2
DA: dopamine
DAPI: 4 ′, 6-Diamidin-2-phenylindol
GABA: gamma-aminobutyric acid
GFAP: glial fibrillary acidic protein
HD: Huntington’s disease
*HTT*: Huntingtin gene
mHTT: mutated huntingtin protein
Iba1: ionized calcium binding adaptor molecule 1
IR: immunoreactive
MSN: medium spiny neurons
ntg: non-transgenic
PET: positron emission tomography
SEM: standard error mean
TH: tyrosine hydroxylase
TSPO: translocator protein 18 kDa
Ub: ubiquitin
VAChT: vesicular acetylcholine transporter.
